# Association of tumor growth rates with molecular biomarker status: a longitudinal study of high-grade glioma

**DOI:** 10.18632/aging.103110

**Published:** 2020-05-09

**Authors:** Ziwen Fan, Yukun Liu, Shaowu Li, Xing Liu, Tao Jiang, Yinyan Wang, Lei Wang

**Affiliations:** 1 Department of Neurosurgery, Beijing Tiantan Hospital, Capital Medical University, Beijing 100070, China; 2 Department of Neuroradiology, Beijing Tiantan Hospital, Capital Medical University, Beijing 100070, China; 3 Department of Pathology, Beijing Tiantan Hospital, Capital Medical University, Beijing 100070, China; 4 Beijing Neurosurgical Institute, Capital Medical University, Beijing 100070, China

**Keywords:** volumetric MRI, high-grade gliomas, tumor growth rate, molecular biomarkers, IDH1

## Abstract

To determine the association of molecular biomarkers with tumor growth in patients with high-grade gliomas (HGGs), the tumor growth rates and molecular biomarker status in 109 patients with HGGs were evaluated. Mean tumor diameter was assessed on at least two pre-surgical T_2_-weighted and contrast-enhancement T_1-_weighted magnetic resonance images (MRIs). Tumor growth rates were calculated based on tumor volume and diameter using various methods. The association of biomarkers with increased or decreased tumor growth was calculated using linear mixed-effects models. HGGs exhibited rapid growth rates, with an equivalent volume doubling time of 63.4 days and an equivalent velocity of diameter expansion of 51.6 mm/year. The WHO grade was an independent clinical factor of eVDEs. *TERT* promoter mutation C250T and *MGMT* promoter methylation was significantly associated with tumor growth in univariable analysis but not in multivariable analysis. Molecular groups of *IDH1*, *TERT,* and 1p/19q and *IDH1* and *MGMT* were independently associated with tumor growth. In addition, tumor enhanced area had a faster growth rate than a tumor entity in incomplete enhanced HGGs (*p* = 0.006). Our findings provide crucial information for the prediction of preoperative tumor growth in HGGs, and aided in the decision making for aggressive resection and adjuvant treatment strategies.

## INTRODUCTION

High-grade gliomas (HGGs; World Health Organization [WHO] grades III and IV) [1] are classified as highly malignant due to their fast growth rates and extreme invasiveness [2]. Standard therapy includes maximum neurosurgical resection and adjuvant therapy. Tumor growth rate directly reflects the radiological expansion of HGGs. Several mathematical models to predict the growth rate of gliomas have been proposed in previous studies [3–5]. Apart from volume-doubling time (VDT), [6] tumor growth can be evaluated using equivalent evolution of the tumor radius or diameter, which construct linear growth models [5, 7, 8]. As reported previously, the median velocity of radial expansion of glioblastomas is 29.6 [8] – 30 mm/year [5]. Slower tumor growth rates have been associated with longer survival times [9]. Compared with survival time, tumor growth rate better reflects the inherent characteristics and radiology expansion of the tumor, as this objective value is not influenced by therapy strategy [10, 11].

Due to their fast growth rates and the need for immediate surgery after diagnosis of HGGs, multiple magnetic resonance imaging (MRI) examinations are not generally performed prior to surgery. The factors that influence the inherent growth dynamics of HGGs therefore remain unclear. A serial assessment of tumor volume can be the only approach to precisely assess tumor growth rates, and the influence of tumor-related biomarkers on such rates might help with such assessments. In the present longitudinal study, we collected data from 109 patients with primary HGGs who underwent preoperative MRIs at least twice, and quantitatively investigated changes in growth rate with different clinical characteristics and molecular biomarkers status.

## RESULTS

### Patient demographics

Table 1 listed the clinical characteristics and the relevant subtypes according to the 2016 WHO classification [1]. A total of 109 patients (66 men and 43 women) were enrolled in the present study. The numbers of patients diagnosed with WHO tumor grade III and IV were 59 and 50, respectively. The median age at the time of tumor detection on the first MRI examination was 48 years (IQR [interquartile range], 35–57 years). All patients received at least twice T_2_-weighted images (T_2_WI) scans before surgery, 20 received ≥3 times, while 56 patients received at least twice contrast-enhanced T_1_-weighted image (CE-T_1_WI) scans. The median interval time between the first and the last preoperative MRI examinations was 41 days (IQR, 22.7–114.8 days). The median initial mean tumor diameter (iMTD) was 38.6 cm^3^ (IQR, 30.0–47.4 mm). Only 44 patients were available to be allocated into the non-not otherwise specified (NOS) subtype because others underwent surgery before 2016 and did not undergo diagnostic molecular testing.

**Table 1:** Clinical characteristics.

**Subtypes**	**Total**	**AO, IDH mt-LOH**	**AA, IDH mt**	**AG, IDH wt**	**AG, NOS ^a^**	**Glioblastoma, IDH mt**	**Glioblastoma, IDH wt**	**Glioblastoma, NOS**
Number	109	7	5	10	37	3	19	28
Age at first MRI examination (years)								
Median (IQR)	48 (35-57)	35 (31.8-44.5)	39 (25.8-62.8)	45.50 (33-60)	48 (33.5-56.3)	49 (41.5-52)	53 (47.3-58)	47 (36.5-57.5)
Gender (Female)	43 (39.5%)	4 (57.1%)	1 (20%)	3 (30%)	18 (48.7%)	2 (66.7%)	8 (42.1%)	7 (25%)
Cortisol therapy	7 (6.4%)	0 (0)	0 (0)	1 (10%)	2 (5.4%)	0 (0)	2 (10.5%)	2 (7.1%)
Contrast-enhancement (CE) type at first MRI examination								
Complete enhanced	34 (31.2%)	1 (14.3%)	1 (20%)	1 (10%)	7 (18.9%)	1 (33.3%)	14 (73.7%)	8 (28.6%)
Incomplete enhanced	20 (18.4%)	1 (14.3%)	2 (40%)	2 (20%)	7 (18.9%)	0 (0)	2 (10.5%)	8 (28.6%)
Unknown ^b^	55 (50.5%)	5 (71.4%)	2 (40%)	8 (80%)	23 (62.2%)	2 (66.7%)	3 (15.8%)	12 (42.9%)
Number of lobes involved								
Median (IQR)	1 (1-2)	1 (1-2)	1 (0.8-3)	1 (0-2)	1 (1-2)	1 (1-1.8)	1 (1-2)	1 (1-2)
Bilateral	8 (7.3%)	0 (0)	0 (0)	1 (10%)	5 (13.5%)	0 (0)	2 (10.5%)	0 (0)
Tumor-edema interface								
Blur	53 (48.6%)	6 (85.7%)	4 (80%)	5 (50%)	20 (54.1%)	2 (66.67%)	5 (26.3%)	11 (39.3%)
Initial mean tumor diameter (iMTD, mm)								
Median (IQR)	38.6 (30-47.4)	31.4 (30.4-44.7)	36.8 (28.9-44.9)	34.05 (28.6-39.4)	41 (32.1-51.1)	40.4 (39.5-60.5)	36.1 (29.7-42.6)	37.6 (28.5-52.1)
Interval time between first and last preoperative MRI examinations (days)								
Median (IQR)	41 (22.7-114.8)	326.4 (132.8-1492.5)	284.4 (176.8-383.5)	29.09 (17.2-37.8)	34.0 (18.8-66)	15.8 (14.5-733)	37.7 (28.6-185.4)	45 (25.5-81.5)
Numbers of MRI examinations								
Median (IQR)	2 (2-2)	2 (2-5.5)	2 (2-3.3)	2 (2-3)	2 (2-2)	2 (2-2)	2 (2-2)	2 (2-2)

Abbreviations: LOH, 1p/19q loss of heterozygosity; NOS, not otherwise specified; wt, wild type; mt, mutation type; AO, anaplastic oligodendroglioma; AA, anaplastic astrocytoma; AG, Anaplastic glioma; IQR, interquartile range.

a. Three patients were oligoastrocytoma, IDH mutation and 1p/19q NOS.

b. High grade gliomas with single CE-T_1_WI at pre-surgery MRI examination.

### Evaluating tumor growth rate

**Figure 1:**
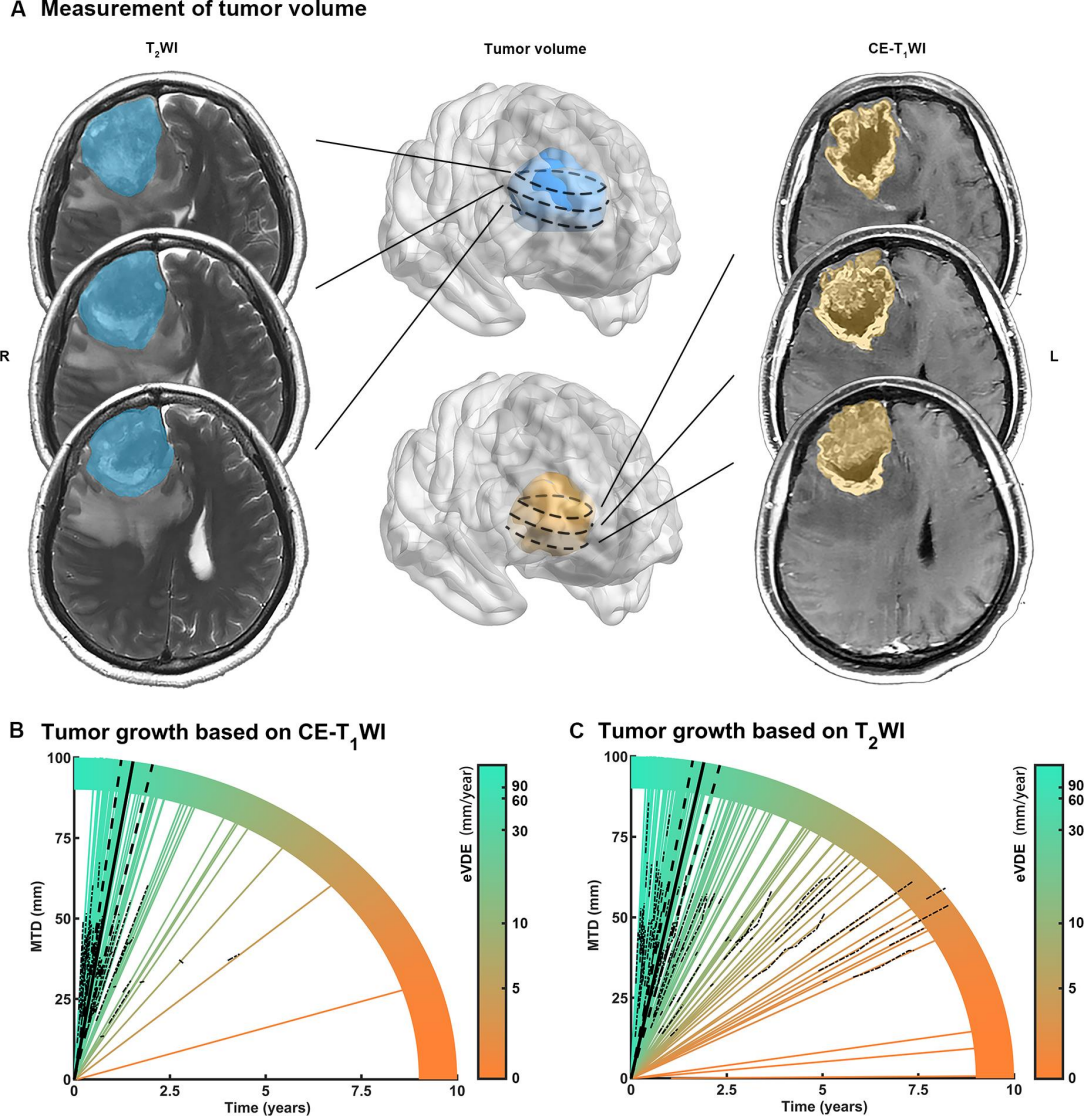
**Measurement of tumor growth rate in high grade gliomas (HGGs).** (**A**) Mean tumor diameter (MTD) was calculated for tumor volume measured by preoperative MR images. (**B**, **C**) Tumor growth trajectories for each patient (colored lines) were aligned with MTD evolution with time (dotted lines). The equivalent velocity of diameter expansions (eVDEs), which represented the slope of tumor growth trajectory, estimated on T2WI (eVDE, 51.6 mm/year; 95% confidence interval [CI], 41.5-61.0 mm/year) and contrast-enhanced T_1_-weighted image (CE-T1WI) (eVDE, 64.3 mm/year; 95% CI, 47.8-90.7 mm/year) were shown in black lines.

Multiple growth rates were calculated based on tumor volume and mean tumor diameter (MTD) (Table 2). The equivalent volume-doubling time (eVDT) of the contrast area (based on CE-T_1_WI) versus tumor entity (based on T_2_WI) was 39.8 days versus 63.4 days, and 61.1 (IQR, 30.8-114.1) versus 40.37 (IQR, 11.7-76) mm/year in median volume-doubling time (VDE). Linear mixed-effects models (LME) with random intercepts was used to evaluate the growth rate of low-grade gliomas (LGGs) and proved that MTD grows linearly [7, 12]. In this study, we considered all the clinical biomarkers as fixed variables and found that iMTD has independent fixed effects (*p* < 0.01, Supplementary Table 1). Thus, for evaluating eVDEs, iMTD was introduced as a fixed variable into LME. Comparing this with the previous method, it was found that the new method had better prediction accuracy than the previous one (*p* < 0.01, Supplementary Table 5) [7, 12]. As for eVDE, tumor entity grew at a speed of 51.6 (95% confidence interval [CI], 41.5-61.0) mm/year, and the enhanced region grew by 64.3 (95% CI, 47.8-80.7) mm/year. The eVDEs for each patient were then fitted and aligned with MTD evolution over time (Figure 1).

**Table 2:** Tumor growth rate estimated in T_2_WI and contrast-enhancement T_1_WI MRI.

	**T_2_WI (N = 109)**		**CE-T_1_WI (N = 54^a^)**	
	**Mean (95% CI)**	**Median (IQR)**	**Mean (95% CI)**	**Median (IQR)**
Absolute volume change (cm^3^)	24.5 (19.2-29.8)	16.1 (4.8-34.1)	22.9 (17.1-28.6)	18.0 (7.6-34.1)
Relative volume change (%)	37.8 (32.9-42.8)	35.0 (18.0-61.5)	54.0 (45.1-62.9)	55.5 (30.0-86.0)
**Volume-based tumor growth rate**				
VDT (days)	274.6 (91.3-457.9)	76.9 (43.6-222.9)	112.1 (42.6-181.6)	46.8 (23.3-101.1)
eVDT (days)	63.4	-	39.8	-
SGR (%)	1.0 (0.8-1.3)	1.0 (0 -1.0)	1.6 (1.3-2.1)	1.7 (0.01-0.3)
**MTD-based tumor growth rate**				
VDE (mm/year)	53.2 (43.1-63.4)	40.37 (11.7-76.0)	75.2 (57.9-92.6)	61.10 (30.8-114.1)
eVDE (mm/year)	51.6 (41.5-61.0)	-	64.3 (47.8-80.7)	-

Abbreviations: CE, contrast-enhanced; CI, confidence interval; IQR, interquartile range; MTD, mean tumor diameter; VDT, volume doubling time; eVDT, equivalent VDT; SGR, specific growth rate; VDE, velocity of diameter expansion; eVDE, equivalent VDE.

a. The other 55 patients received a single CE- T_1_WI MRI before surgery.

### Association of tumor growth rate with clinical and molecular biomarkers status

**Figure 2:**
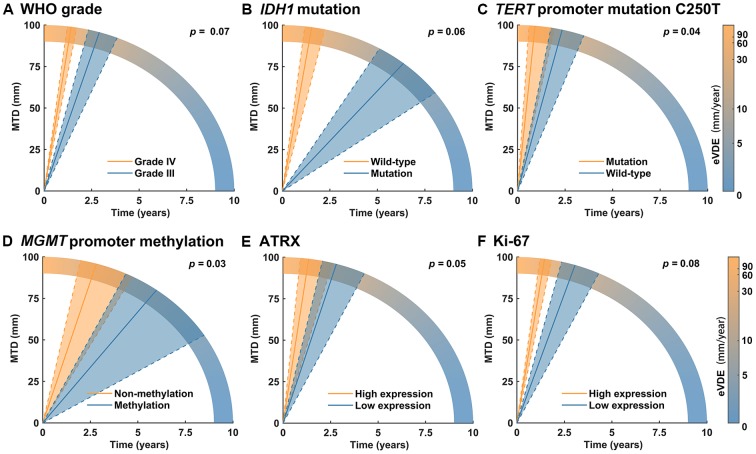
**The association of molecular biomarkers and tumor growth.** (**A**) The WHO grade was marginally significantly associated with tumor growth in multivariable analysis (*p* = 0.07). (**B**–**F**) *TERT* promoter mutation C250T (*p* = 0.04), ATRX (*p* = 0.05) and Ki-67 high expression (*p* = 0.08), *IDH1* mutation (*p* = 0.06) and *MGMT* promoter methylation (*p* = 0.03) showed significant or marginally significant association with tumor growth in univariable analysis but not in multivariable analysis (*p* > 0.05).

To avoid confounding the cohort effects of the clinical biomarkers, we first separately introduced each biomarker as an interaction term and then introduced the significant ones together into interaction terms. Only the WHO grade was an independent factor of the clinical biomarkers, and higher grade (WHO grade IV) was associated with increased tumor growth rate (+27.5 ± 9.8 mm/year, *p =* 0.005, Supplementary Table 2). having introduced iMTD as a fixed effect and the WHO grade as an interaction term, the molecular biomarkers were then introduced separately as interaction terms into the univariable analysis. As a result, *telomerase reverse transcriptase* (*TERT*) promoter mutation C250T (+ 52.4 ± 25.7 mm/year, *p* = 0.04) was significantly associated with increased tumor growth, while *O-6-methylguanine-DNA methyltransferase (MGMT)* promoter methylation (-37.4 ± 17.6 mm/year, *p* = 0.03) was significantly associated with decreased tumor growth. High α-thalassemia X-linked intellectual disability (ATRX) expression (+ 31.6 ± 16.2 mm/year, *p* = 0.05, score 3/4 versus 0-2); Ki67 expression (+ 20.1 ± 11.3 mm/year, *p* = 0.08; score 3/4 versus 0-2); and *isocitrate dehydrogenase 1 (IDH1)* mutation (-28.7 ± 15 mm/year, *p* = 0.06) were marginally significantly associated with tumor growth (Figure 2, the results of other molecular biomarkers are shown in Supplementary Table 3). These significant and marginally significant biomarkers were introduced together as interaction terms into multivariable analysis, and the results exhibited that only the WHO grade (+19.1 ± 10.5, *p* = 0.07) showed marginal independence (Table 3).

**Table 3:** Univariable and multivariable linear mixed-effects model for the association of biomarkers with tumor growth rate.

**Biomarker**	**Group 1**		**Group2**			**Univariable analysis^a^**				**Multivariable analysis^b^**		
	**N**	**eVDE (95% CI)**	**N**	**eVDE(95% CI)**		**Interaction coefficients**	**SE**	***p*-value**		**Interaction coefficients**	**SE**	***p*-value**
WHO grade Grade IV vs III	56	69.3 (54.9-83.7)	55	32.9 (22.3-43.6)		+27.5	9.8	0.005**		+19.1	10.5	0.07*
*IDH1* mt vs wt	18	12.1 (6.4-17.7)	29	63.9 (39.8-88.1)		-28.7	15	0.06*		-18.5	16.6	0.3
*MGMT* promoter met vs non-met	27	13.4 (4.7-22.0)	10	34 (15.7-52.3)		-37.4	17.6	0.03**		-23.9	18.6	0.2
*TERT* C250T promoter mt vs wt	4	107.7 (50.4-165)	37	41.4 (24.0-58.8)		+52.4	25.7	0.04**		+32.1	26.8	0.2
ATRX high vs low expression	14	73.3 (29.8-116.7)	21	34.2 (18.5-49.8)		+31.6	16.2	0.05*		+24	17.3	0.2
Ki67 high vs low expression	55	67.8 (51.3-84.3)	36	31.1 (18.9-43.3)		+20.1	11.3	0.08*		+13.8	11.4	0.2

Abbreviations: wt, wild type; mt, mutation type; met, methylation; SE, standard error.

a. A single molecular biomarkers plus significant clinical biomarkers in linear mixed-effects model.

b. Significant clinical and molecular biomarkers in linear mixed-effects model together.

* *p*-value < 0.1 showed marginally statistically significance.

** *p*-value < 0.05 showed statistically significance.

To further investigate the association of molecular biomarkers with tumor growth, combinations of molecular biomarkers were introduced into interaction terms. After adjusting for WHO grade, ATRX, Ki67, and *MGMT*, the *IDH1*, *TERT,* and 1p/19q molecular groups were independently associated with tumor growth. HGGs with *TERT* promoter mutation (C250T or C228T) only had a significantly faster growth rate than other groups. After adjustment for WHO grade, ATRX, Ki67 and *TERT*, the molecular groups of *IDH1* and *MGMT* were independently associated with tumor growth. HGGs with *IDH1* wild type and *MGMT* promoter methylation had a significantly faster growth rate than other groups. After adjustment for WHO grade, *MGMT*, Ki67, and *TERT,* molecular group of *IDH1* and ATRX were associated with tumor growth in univariable analysis but not in the multivariable model. (Table 4, details of the WHO grade and other molecular biomarkers are shown in Supplementary Table 4).

**Table 4:** Univariable and multivariable linear mixed-effects model for the association of combined molecular groups with tumor growth rate.

**Molecular groups**	**No.**	**Univariable analysis**				**Multivariable analysis**		
		**Interaction coefficients**	**SE**	***p***		**Interaction coefficients**	**SE**	***p***
*IDH1* + *TERT* +1p/19q^a^								
Triple-positive	6	reference				reference		
*TERTmt* and *IDH1mt*	3	0.8	30.6	0.98		0.3	31.2	0.99
*IDH1*mt only	5	32.0	27.7	0.3		27.0	30.6	0.4
*TERT*mt only	12	73.0	23.2	0.002*		64.4	26.4	0.02*
Triple-negative	12	18.5	22.8	0.4		17.7	25.4	0.5
*IDH1 + MGMT^b^*								
*IDH1*mt and *MGMT*met	14	reference				reference		
*IDH1*mt/*MGMT*met only	14	29.4	18.0	0.1		34.3	19.7	0.08
*IDH1* wt and non-*MGMT*met	9	61.0	20.9	0.004*		57.8	21.2	0.01*
*IDH1 +* ATRX^c^								
*IDH1mt* + ATRX low	7	reference				reference		
*IDH1mt* + ATRX high	5	-10.4	25.9	0.7		-14.4	26.8	0.6
*IDH1wt* + ATRX low	14	3.3	21.5	0.9		-3.1	22.8	0.9
*IDH1wt* + ATRX high	9	61.9	23.7	0.01*		48.8	25.9	0.06

Abbreviations: wt, wild type; mt, mutation type; met, methylation; SE, standard error.

a. After adjustment for WHO grade, ATRX, Ki67 and *MGMT*.

b. After adjustment for WHO grade, ATRX, Ki67 and *TERT*.

c. After adjustment for WHO grade, *MGMT*, Ki67 and *TERT*.

* *p*-value < 0.05 showed statistically significance.

### Comparison of the growth rate in different contrast enhancement types

**Figure 3:**
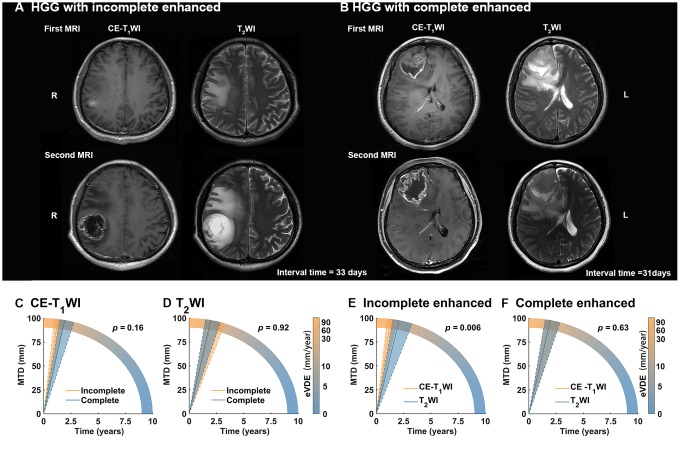
**Tumor growth rate in different contrast enhancement (CE) type.** (**A**, **B**) The longitudinal contrast-enhanced T_1_-weighted image (CE-T_1_WI) and T_2_WI MR-images with incomplete enhanced and complete enhanced high-grade gliomas (HGGs), respectively. (**C**, **D**) For HGGs with two or more CE- T_1_WI MR images (n = 54), equivalent velocity of diameter expansions (eVDEs) in different CE type based on T_2_WI (represented tumor entity) and CE-T_1_WI (represented tumor enhanced area) showed no significant difference (*p* > 0.05). (**E**, **F**) HGGs with incomplete enhanced showed significant faster eVDEs in tumor enhanced area than tumor entity (*p* = 0.006). However, HGGs complete enhanced showed no significant difference in eVDEs between tumor enhanced area and tumor entity (*p* = 0.63).

eVDEs were compared in patients with at least twice CE-T_1_WI scans (n = 54) (Figure 3). Firstly, eVDEs of the complete enhanced HGGs and incomplete HGSSs were compared based on CE-T_1_WI (tumor enhanced area) and T_2_WI (tumor entity), respectively. The results showed no significant difference in eVDEs in contrast to the enhanced type (*p* > 0.05). Then, eVDEs of the tumor enhanced area and tumor entity were compared in different contrast enhancement types. We found that tumor enhanced area (75.4, 90% CI, 51.8-99 mm/year) showed significantly higher growth rate than tumor entity in incomplete enhanced HGGs (46.7, 90% CI, 28.6-64.8 mm/year, *p* = 0.006); however, there was no significant difference in complete enhanced HGGs (*p >* 0.05).

## DISCUSSION

The tumor growth rate directly reflects the radiological expansion of HGGs, and avoided the influence of surgical treatment or adjuvant therapy. Currently, there is no quantitative measurement of the change in tumor growth associated with HGG genetic characteristics. In the current study, we measured tumor growth rates in 109 HGG patients and identified the quantitative change in tumor growth rates associated with clinical and molecular biomarkers. The WHO grade was an independent clinical factor of eVDEs. *TERT* promoter mutation C250T and *MGMT* promoter methylation were significantly associated with tumor growth in univariable analysis but not in multivariable analysis. Molecular groups of *IDH1*, *TERT,* and 1p/19q; and *IDH1* and *MGMT* were independently associated with tumor growth. In addition, tumor enhanced area had a faster growth rate than tumor entity in incomplete enhanced HGGs.

Tumor growth rate acts as an intrinsic indicator of the tumor’s biological behavior. Lower growth rates have always been associated with better prognosis [9]. Previous studies have shown that tumor growth kinetics fit a Gompertzian growth model based on tumor volume, and a linear growth model based on tumor diameter [3–5]. VDT has been used to represent tumor growth rate in most previous studies [13, 14], with a mean VDT for glioblastomas ranging from 9.7–95 days [15]. In addition, the specific growth rate (SGR) and eVDT for glioblastomas were reported to be 1.4% and 49.6 days, respectively. In previous studies, the velocities of glioblastoma radius expansion was reported to be 29.6 [8] – 30 mm/year [5]. Because we measured the velocity of expansion based on the diameter, our results exhibited a eVDE of 69.3 mm/year for glioblastomas, and a eVDE of 51.6 mm/year for all HGGs.

The LME is suitable for measuring tumor growth rate. In general, neuroimaging data exhibit the following distinctive longitudinal data characteristics [16, 17]: (1) longitudinal measurement over time at multiple time-points from the same cohort, reflect the temporal trajectory of measurements; (2) due to the costs and complexities of data collection, longitudinal designs were imbalanced at varying time-points, and most patients had only two measurement time-points and time intervals; (3) the inter-subject variability makes classic regression approaches inappropriate for such data, and they may increase as a function of time due to the diverging trajectories of the individuals in the cohort. By combining the fixed effects, LME fitted these characteristics with greater precision of measurement and without the confounding cohort effects, compared with the mean temporal trajectory [16, 18]. Additionally, based on the random effects, LME allowed for the evaluation of imbalanced longitudinal data and separate analysis of between- and within-subject variability. As a result of the random effect introduction, our model showed better prediction accuracy than the LME method used in previous studies (Supplementary Table 5) [7, 12].

While the qualitative influence of the clinical and molecular biomarkers on tumor growth rate has been extensively investigated [19–24], their quantitative influence on tumor behavior has not been thoroughly studied. Thus, we quantitatively analyzed various clinical and molecular biomarkers to evaluate their association with tumor growth. WHO tumor grade was an independent clinical factor while higher WHO grade was associated with increased tumor growth. We observed faster growth rate in the contrast-enhanced areas than on the entire tumor. Furthermore, we found that incomplete enhanced HGGs had a significantly higher growth rate in enhanced tumor areas than tumor entity. We proposed a possible explanation that the speed of the disrupted blood-brain barrier (BBB) was faster than that of tumor entity growth [25]. For the complete enhanced tumors, with the BBB disrupted completely, the speed of the BBB disruption was limited by the tumor growth so that the result showed no significant difference between tumor entity and enhanced area.

In addition to the clinical biomarkers, univariable analysis, which introduced the WHO grade as an additional interaction term and thus excluded its effects on tumor growth, suggested that the status of *MGMT, TERT, and* C250T were associated with tumor growth rate. In addition, *IDH1* mutation and low expression of ATRX and Ki67 showed marginally significant association with decreased tumor growth. The association of those molecular biomarkers and tumor growth may confer a prognostic value to the molecular biomarkers [26–34]. However, molecular biomarkers that were significantly or marginally significantly associated with tumor growth in univariable analysis showed no significant association in multivariable analysis. Previous study showed that the combination of *MGMT* and *IDH1* showed better prediction performance than *MGMT* alone [35]. *TERT* mutation was also highly associated with I*DH1* type in the prediction of prognosis [15, 36, 37]. Thus, considering the prognostic implications of different combinations of molecular biomarker groups, we defined several molecular groups to further investigate their association with tumor growth. Molecular groups of *IDH1*, *TERT,* and 1p/19q; and *IDH1* and *MGMT* were independently associated with tumor growth. In the first molecular group, *TERT* mutation only (*IDH1* wild type, non-1p19q loss of heterozygosity [LOH]) showed the highest growth rate than other types, which conformed with previous study findings that this molecular group showed the worst prognosis in HGGs [37]. In the latter group, *IDH1* wild type and *MGMT* promoter non-methylation also showed the highest growth rate as well as the worst prognosis in previous studies [35, 38]. The *IDH1* and ATRX molecular group was associated with tumor growth in univariable analysis but not in multivariable analysis, which only demonstrated a marked separation in survival in the astrocytoma with 1p/19q LOH and *IDH* mutation [30].

There are several limitations to the present study. Given the retrospective nature of this study, the number of available molecular biomarkers for each patient varied. Thus, the samples used in the analysis for each biomarker were inconsistent, which also limited the power of the multivariable analysis. Therefore, a larger independent glioma dataset that includes comprehensive genomics data should be used to validate our results and further reveal the associations between genetic characteristics and glioma growth.

In conclusion, we investigated the rapid growth rate of HGGs and the quantitative change in tumor growth rates associated with the clinical and molecular biomarker status. Considering the imbalanced longitudinal data and variations between individuals, LME was used, which provided a parsimonious way to represent the group mean temporal trajectory of the measurements. The growth rate of HGGs was 51.6 mm/year, calculated in eVDE. The WHO grade was an independent clinical factor of tumor growth. In univariable analysis, *TERT* promoter mutation C250T was significantly associated with increased tumor growth (+ 52.4 mm/year), while *MGMT* promoter methylation was significantly associated with decreased tumor growth (-37.4 mm/year). All the molecular biomarkers that were significantly or marginally significantly associated with tumor growth in univariable analysis, showed no significant association with tumor growth in multivariable analysis. The *IDH1*, *TERT,* and 1p/19q; and *IDH1* and *MGMT* molecular groups were independently associated with tumor growth. In addition, tumor enhanced area had a faster growth rate than tumor entity in incomplete enhanced HGGs. Such findings may assist clinicians in planning for an aggressive surgical resection and adjuvant treatment, and may aid in the clinical prediction of tumor growth rates even after surgery, based on the tumor-related biomarkers.

## MATERIALS AND METHODS

### Patient selection

We retrospectively reviewed the clinical information and imaging data from patients with gliomas who underwent primary surgical treatment between January 2008 and March 2019. The inclusion criteria were as follows: 1) age ≥18 years at diagnosis, 2) two or more MRI examinations were performed prior to surgery, 3) no chemotherapy or radiotherapy was administered prior to surgery, and 4) WHO grade III or IV glioma was confirmed histopathologically. To avoid bias, patients for whom sequential MRIs were performed at intervals of less than 14 days were excluded from this study. A total of 109 patients with HGGs were finally included.

### Standard protocol approvals, registrations, and patient consents

All clinical information was retrospectively collected from the institutional medical database and the retrospective analysis of this study was approved by the local institutional review board.

### Magnetic resonance imaging data acquisition

For most patients, MRI scans were obtained using a Magnetom Trio 3T scanner (Siemens AG, Erlangen, Germany). In other cases, imaging data were acquired using a Magnetom Verio 3T scanner (Siemens AG, Erlangen, Germany). T_2_WI were obtained with the following imaging parameters: TR = 5800 ms; TE = 110 ms; field of view = 240188 mm^2^; flip angle = 150°; and voxel size = 0.6×0.6×5 mm^3^. Gadopentetate dimeglumine (Ga-DTPA injection, Beijing, Beilu Pharma) was injected intravenously at a dose of 0.1 mM/ kg, and post-contrast T1-weighted images were collected after contrast injection. T1-weighted images were obtained with the following parameters: TE = 15 ms, TR = 450 ms, and slice thickness = 5 mm. The contrast-enhanced area included the contrast area and the necrotic region, marked on the CE-T_1_WI. The brain lesions of each patient were manually segmented by two neurosurgeons using the free access software MRIcro (http://www.mccauslandcenter.sc.edu/mricro/), a senior neuroradiologist determined the lesion border if a discrepancy of more than 5% was observed.

### IDH1 mutations and MGMT promoter methylation

*IDH1* mutations were identified using DNA pyrosequencing [36]. In brief, a QIAamp DNA Mini Kit (Qiagen) was used to isolate genomic DNA from frozen tumor tissue samples. The genomic region spanning the wild-type R132 of *IDH1* was analyzed by amplifying a 75-base pair (bp) fragment with the following primers: 5′-GCTTGTGAGTGGATGGGTAAAAC-3′ and 5′-biotin-TTGCCAACATGACTTACTTGATC-3′. Duplicate PCR analyses were performed in 40 μL reaction tubes containing 1 μL each of 10 μM forward and reverse primers, 4 μL of 10 × buffer, 3.2 μL of 2.5 mM dNTPs, 2.5 U HotStar Taq (Takara), and 2 μL of 10 μM DNA. The PCR conditions were as follows: 95°C for 3 minutes, 50 cycles at 95°C for 15 seconds, 56°C for 20 seconds, 72°C for 30 seconds, and then 72°C for 5 minutes (ABI PCR System 9700; Applied Biosystems). Single-stranded DNA was purified from the PCR products and pyrosequenced with a PyroMark Q96 ID System (Qiagen) using a 5′-TGGATGGGTAAAACCT-3′ primer and an EpiTect Bisulfite Kit (Qiagen).

The methylation status of the *MGMT* promoter was also detected using DNA pyro-sequencing as previously reported [15, 39].

### TERT promoter mutation

Mutations of the *TERT* promoter were identified by polymerase chain reaction (PCR) and Sanger sequencing [15]. Sequences covering genomic mutational hotspots in the *TERT* core promoter region (nucleotide positions 1,295,228 [C228T] and 1,295,250 [C250T]) were amplified using nested PCR with reference to the human genome reference sequence (grCh37 February 2009; http://genome.ucsc.edu/). PCR was carried out in a total volume of 10 μl containing 1 μl DNA (10–50 ng/μl), Platinum Taq DNA polymerase (1 unit), 1 μl of 10X PCR buffer (Invitrogen, Carlsbad, CA, USA), 1.0 mM MgCl_2_, 0.1 mM of each dNTP, 1% (v/v) dimethyl sulfoxide, and 0.25 mM of each primer. Amplified products were purified using the Illustra ExoProStar system (GE Healthcare, Buckinghamshire, UK) to remove any unused primer and were then subjected to direct sequencing with a BigDye Terminator cycle sequencing kit (Applied Biosystems, Foster City, CA, USA) on an ABI 3100 PRISM DNA sequencer. Before sequencing, the quality of all PCR products was checked via electrophoresis on 2% agarose gels.

### Detection of 1p/19q codeletion

Representative tumor areas were marked on hematoxylin and eosin-stained sections. The corresponding areas were identified on paraffin blocks, and new 4 μm sections were prepared. The material was deparaffinized with xylene, incubated with 0.3% pepsin in 10 mM HCl at 37°C for 10 minutes, and denatured at 85°C for 10 minutes. Dual-color fluorescence in situ hybridization was performed using LSI probe sets for 1p36/1q25 and 19q13/19p13 (spectrum orange-labeled 1p36 and 19q13 probes; spectrum green-labeled 1q25 and 19p13 probes; and Vysis) and evaluated in at least 200 non-overlapping nuclei with intact morphology.

### Immunohistochemical staining

The details of the immunohistochemistry performed was included in the supplementary files. Briefly, formalin-fixed tumor tissues were dehydrated in ethanol and embedded in paraffin. Five-micron-thick sections were prepared, and immunohistochemical staining was performed using antibodies from Zhongshan Gold Bridge Biotechnology of ATRX (1:100 dilution; ZA-0016), primary glial fibrillary acidic protein (GFAP; 1:100 dilution; ZM-0118), oligodendrocyte transcription factor (Olig-2; 1:100 dilution; ZA-0561), topoisomerase II (TOPO2; 1:100 dilution; ZM-0245), P170 (1:100 dilution; ZM-0189), matrix metallopeptidase 9 (MMP9; 1:100 dilution; ZA-0562), glutathione S-transferase π (GST-π; 1:100 dilution; ZM-0110), Ki67(1:100 dilution; ZM-0167), MGMT(1:100 dilution; ZM-0461), epidermal growth factor receptor (EGFR; 1:100 dilution; ZA-0505), vascular endothelial growth factor (VEGF; 1:100 dilution; ZA-0509), phosphatase and tensin homolog (PTEN; 1:100 dilution; ZA-0635) and p53 (1:100 dilution; ZM-0408), according to the protocols.

For the histopathological scoring, the sections were reviewed by two neuropathologists who were blinded to the clinical data. Staining was scored on a 5-point scale ranging from 0 to 4 as follows: 0 = no or rare occurrence of staining, 1 = 10% of cells positively stained, 2 = 10-30% of cells positively stained, 3 = 30-60% of cells positively stained, and 4 = over 60% of cells positively stained. To obtain a sample size of the subgroups that would meet the statistical requirements and determine a meaningful segmentation point of dichotomy for each biomarker, cutoffs were defined. Pathologists who conducted the immunohistochemical analyses were blinded to the clinical and molecular information.

### Assessment of inherent tumor growth

The tumor volume (*V*) was calculated based on the segmented tumor region drawn on transverse T_2_WI (Figure 1) using MATLAB (version 2014a, The MathWorks Inc., MA, USA). The contrast-enhanced area was based on CE-T_1_WI. The growth rate of HGGs can be assessed using VDT, SGR, eVDT, velocity of diameter expansion (VDE), and equivalent VDE (eVDE). VDT was calculated based on tumor volume [(VDT = Δ*T* × log 2/(log*V*_2_ – log*V*_1_), where *V*_1_ represents the tumor volume at the first MRI examination, *V*_2_ represents the tumor volume at the most recent MRI examination prior to surgery, and Δ*T* represents the time interval]. SGR was calculated based on VDT (SGR = ln 2/*VDT*), and eVDT was calculated using mean SGR (eVDT = ln 2/*SGR*). SGR is considered to yield a highly symmetrical distribution, while eVDT is considered to yield a more precise estimate of the true growth rate in the population than the median VDT. In addition, VDE was estimated by the linear regression of MTD, (MTD = (2 × *V*)^1/3^) for each patient over time [11].

To characterize changes in MTDs over time and their association with clinical and molecular biomarkers, LMEs were used for the longitudinal data [16, 40]. LME provided more precise predictions of MTD evolution over time and without confounding cohort effects. We used the following formula in this study:

$$MTD_{ij}=\beta_{0}+\beta_{1}\times T_{ij}+\beta_{m}\times I_{m}+\alpha_{1i}+\alpha_{2i}\times T_{ij}+\varepsilon_{ij}$$

*MTD_ij_* denotes the MTD for patient *i* at time of observation *j*. *I* = (*I*_1_, *I*_2_, … *I_m_*) are the fixed effects of biomarkers. *α* and *β* represent the coefficients of random effects and fixed effects, respectively. *β*_1_ represents eVDE. *T_ij_* represents the time of observation *j* from first observation for patient *i*. *ε_ij_* is the residual term. Considering the inter-patient variations, fitted eVDE for patients *i* can be described as (*β*_1_ + *α*_2*i*_).

Biomarkers with significant fixed effects were introduced in LME. To describe the change in eVDE associated with the status of *I* = (*I*_1_, *I*_2_, … *I_n_*), interaction terms were introduced in LME, as presented below:

$$\begin{array}{c} MTD_{ij}=\beta_{0}+\beta_{1}\times T_{ij}+\beta_{m}\times I_{m}+\beta_{n}\times (I_{n}\times T_{ij})+\beta_{n+1}\times I_{n}\\ +\alpha_{1i}+\alpha_{2i}\times T_{ij}+\varepsilon_{ij} \end{array}$$

Thus, the change in eVDE (increased or decreased) associated with the factor *I_n_* are represented as *β_n_*.

### Statistical analyses

Statistical analysis was conducted using MATLAB 2014a. To select the fixed variables represented by the significant association with MTD, clinical biomarkers such as age, gender, WHO grade, iMTD, interval time between the first and last MRI examinations, number of MRI examinations, cortisol therapy, contrast enhancement type, tumor-edema interface (clear or blur), number of lobes involved and brain structures involved (frontal lobe, parietal lobe, occipital lobe, temporal lobe, insular lobe, stem, thalamus, cerebellum and ventricle) were introduced, separately into LME. Then the clinical biomarkers with significant fixed effect were introduced together, and fixed variables were selected from significant biomarkers. To select the clinical variables that were significantly associated with eVDE, clinical biomarkers were then introduced into an interaction term alone with time, and then those with significant interaction coefficients were introduced together. Thus, we found the independent clinical biomarkers associated with eVDEs. These variables and time, along with single molecular biomarkers were then introduced into interaction terms in univariable analysis to determine the significance of the molecular biomarkers associated with tumor growth. To find independent factors of eVDEs, we introduced all the significant and marginally significant biomarkers into the interaction terms in multivariable analysis.

For biomarkers with unknown or NOS group, we first evaluated the NOS group with the non-NOS group to determine whether the subgroups were distributed inconsistently between those two groups. Only biomarkers with consistent distribution were taken into consideration. Molecular biomarkers, examined by immunohistochemical staining were allocated to different subgroups according to their expression levels (low versus high, the cutoff was selected by the significant *p*-values, Supplementary Table 3). A *p* value < 0.05 was considered to be significant and *p* value between 0.05 and 0.1 was considered to be marginally significant [41, 42].

### Data availability statement

Anonymized data will be shared by request from any qualified investigator.

## Supplementary Material

Supplementary Materials and Methods

Supplementary Tables 1-2, 4-6

Supplementary Table 3
