# Supplementary material for: Association of tumor growth rates with molecular biomarker status: a longitudinal study of high-grade glioma
Source: Aging (Albany NY). 2020 May 9;12(9):7908–26. doi: 10.18632/aging.103110 (PMC7244074; doi:10.18632/aging.103110)
Supplement: Supplementary Materials and Methods [file aging-12-103110-s002..pdf]

SUPPLEMENTARY MATERIALS AND METHODS

Supplementary details of immunohistochemical procedure

Surgical specimens of high-grade gliomas (HGGs) were fixed in formalin, dehydrated in ethanol, routinely processed and paraffin embedded. Five-micron-thick sections were prepared and dried at 80°C for 15 min. They were then dewaxed in xylene, rinsed in graded ethanol and rehydrated using double-distilled water. To block the endogenous peroxidase activity, the sections were treated with 3 % H_2O_2 (Beijing Chemistry) for 5 min at room temperature (25 °C). For antigen retrieval, sections were steamed in sodium citrate buffer (10 mM sodium citrate, pH 6.0) (Beijing Chemistry) for 15 min at 100 °C. Then, sections were washed in phosphate-buffered saline for 3 min and blocked with 5% bovine serum albumin for 1 h at 37 °C. The sections were immunostained with the antibodies from Zhongshan Gold Bridge Biotechnology (details were shown in Supplementary Table 6), such as α-thalassemia X-linked intellectual disability (ATRX; 1:100 dilution; ZA-0016), primary glial fibrillary acidic protein (GFAP; 1:100 dilution; ZM-0118), oligodendrocyte transcription factor (Olig-2; 1:100 dilution; ZA-0561), topoisomerase II (TOPO2; 1:100 dilution; ZM-0245), P170 (1:100 dilution; ZM-0189), matrix metallopeptidase 9 (MMP9; 1:100 dilution; ZA-0562), glutathione S-transferase π (GST-π; 1:100 dilution; ZM-0110), Ki67(1:100 dilution; ZM-0167), MGMT(1:100 dilution; ZM-0461), epidermal growth factor receptor (EGFR; 1:100 dilution; ZA-0505), vascular endothelial growth factor (VEGF; 1:100 dilution; ZA-0509), phosphatase and tensin homolog (PTEN; 1:100 dilution; ZA-0635) and p53 (1:100 dilution; ZM-0408), and were incubated at 4 °C overnight. After being washed in PBS, the tumor samples were incubated in the secondary antibody for 30 min at room temperature. The sections were washed again and treated with Elite ABC (Vectastain; Vector Laboratories, Burlingame, CA, USA), and washed and developed with 3,3′diaminobenzidine tetrahydrochloride (50 mg 3,3′-diaminobenzidine and 150 µl 3% H_2O_2 in 100 ml PBS). After being rinsed in PBS, the samples were dehydrated in graded alcohols, cleared in xylene, and permanently covered. The sections were analyzed by light microscope (Nikon ECLIPSE 80i). To ensure that the semi-quantification of the IHC staining in the images was performed properly, 10 fields of view were randomly selected under high power fields (magnification × 200), and average proportion of positively stained tumor cells was evaluated.

Controls without primary antibody and positive control tissues were included in all experiments to ensure the quality of staining.
