# Supplementary material for: Association of tumor growth rates with molecular biomarker status: a longitudinal study of high-grade glioma
Source: Aging (Albany NY). 2020 May 9;12(9):7908–26. doi: 10.18632/aging.103110 (PMC7244074; doi:10.18632/aging.103110)
Supplement: Supplementary Tables 1-2, 4-6 [file aging-12-103110-s003..pdf]

SUPPLEMENTARY TABLES

Supplementary Table 1. Univariable and multivariable linear mixed-effect model for the association of clinical biomarkers with mean tumor diameter (MTD).

Fixed effects [a]	Univariable analysis				Multivariable analysis			
I_m	β_m	SE	p	p of ANOVA[b]	β_m	SE	p	p of ANOVA
Initial MTD	1.0	0.01	< 0.01**	—	1.0	0.01	< 0.01**	—
WHO grades					—	—	—	—
Grade IV vs III	-0.74	2.69	0.84	—	—	—	—	—
Interval time	-0.004	0.003	0.20	—	—	—	—	—
Number of MRI examinations	-0.78	1.16	0.53	—	—	—	—	—
Age	-0.14	0.1	0.13	—	—	—	—	—
Gender					—	—	—	—
Male vs female	6.09	2.69	0.02**	—	-0.19	0.22	0.37	
Cortisol history	-10.83	5.38	0.05*	—	0.19	0.36	0.61	
Contrast-enhanced type				0.03**	—	—	—	0.81
Complete vs incomplete	-8.6	3.15	0.01**	—	-0.13	0.4	0.74	—
Unknown vs non-unknown [c]	3.1	2.69	0.25	—	—	—	—	—
Tumor-edema interface								
Clear vs blur	-5.16	2.65	0.05*	—	0.18	0.26	0.49	—
Brain side				0.79				
Right Side vs Left Side	1.85	2.81	0.51	—	—	—	—	—
Bilateral vs Left Side	0.02	5.34	0.99	—	—	—	—	—
Number of lobes involved	7.95	1.24	< 0.01**	—	-0.05	0.17	0.78	—
Frontal lobe involved	2.74	2.72	0.32	—	—	—	—	—
Parietal lobe involved	9.44	2.79	< 0.01**	—	0.03	0.28	0.93	—
Occipital lobe involved	11.62	3.86	<0.01**	—	0.14	0.34	0.68	—
Temporal lobe involved	9.17	2.55	< 0.01**	—	0.01	0.25	0.96	—
Insular lobe involved	0.47	3.62	0.9	—	—	—	—	—
Stem involved	0.18	5.16	0.97	—	—	—	—	—
Thalamus involved	4.03	5.47	0.46	—	—	—	—	—
Cerebellum involved	-4.20	7.14	0.56	—	—	—	—	—
Ventricle involved	-4.01	6.42	0.53	—	—	—	—	—

Abbreviation: WHO, World Health Organization; SE, standard error; ANOVA, analysis of variance; LME, linear mixed-effect model.

a. LME in prediction of MTD:

$$MTD_{ij} = \beta_0 + \beta_1 \times T_{ij} + \beta_m \times I_m + \alpha_{1i} + \alpha_{2i} \times T_{ij} + \varepsilon_{ij}$$

b. Biomarkers with more than 2 classification types were used ANOVA to estimate their fixed effects on MTD.

c. For biomarkers with unknown or NOS group, we first evaluate the NOS group with the non-NOS group to figure out if the subgroups were distributed inconsistency between those two groups. Only biomarkers with consistent distribution were taken into multivariable analysis.

* p-value < 0.1 showed marginally statistically significance.

** p-value < 0.05 showed statistically significance.

Supplementary Table 2. Univariable and multivariable linear mixed-effect model for the association of clinical biomarkers with tumor growth.

Interaction term[a]	Univariable analysis				Multivariable analysis			
I_n	β_n	SE	p	p of ANOVA[b]	β_n	SE	p	p of ANOVA
Initial MTD	0.19	0.35	0.60	—	—	—	—	—
WHO grades								
Grade IV vs III	34.37	9.24	< 0.01**	—	27.48	9.77	0.01**	—
Interval time	-0.03	0.01	0.01**	—	-0.02	0.01	0.19	—
Number of MRI examinations	-8.35	3.01	0.01**	—	-6.08	4.31	0.16	—
Age	0.63	0.35	0.08*	—	0.17	0.38	0.65	—
Gender								
Male vs female	2.56	10.09	0.80	—	—	—		—
Cortisol history	15.13	19.92	0.45	—	—	—		—
Contrast-enhanced type				0.13				—
Complete vs incomplete	-4.07	1.84	0.03	—	—	—	—	—
Unknown vs non-unknown [c]	16.28	9.40	0.08	—	—	—	—	—
Tumor-edema interface								—
Clear vs blur	20.12	8.45	0.02**	—	-2.24	10.68	0.83	
Brain side				0.11				—
Right Side vs Left Side	15.28	9.12	0.10	—	—	—		—
Bilateral vs Left Side	-15.05	17.40	0.39	—	—	—	—	—
Number of lobes involved	4.62	4.72	0.33	—	—	—	—	—
Frontal lobe involved	-10.24	8.81	0.25	—	—	—	—	—
Parietal lobe involved	19.46	9.48	0.04**	—	-24.91	18.22	0.17	—
Occipital lobe involved	29.36	14.55	0.04**	—	8.50	10.57	0.42	—
Temporal lobe involved	7.36	8.84	0.41	—	—	—	—	—
Insular lobe involved	0.71	11.29	0.95	—	—	—	—	—
Stem involved	-36.19	18.48	0.05*	—	8.92	14.97	0.55	—
Thalamus involved	2.94	20.67	0.89	—	—	—	—	—
Cerebellum involved	-5.02	22.75	0.83	—	—	—	—	—
Ventricle involved	5.13	22.69	0.82	—	—	—	—	—

Abbreviation: WHO, World Health Organization; SE, standard error; ANOVA, analysis of variance; LME, linear mixed-effect model.

a. Evaluation of clinical acceleration effects in LME:

$$MTD_{ij} = \beta_0 + \beta_1 \times T_{ij} + \beta_2 \times iMTD + \beta_n \times (I_n \times T_{ij}) + \beta_{n+1} \times I_n + \alpha_{1i} + \alpha_{2i} \times T_{ij} + \varepsilon_{ij}$$

b. Biomarkers with more than 2 classification types were used ANOVA to estimate their fixed effects on MTD.

c. For biomarkers with unknown or NOS group, we first evaluate the NOS group with the non-NOS group to figure out if the subgroups were distributed inconsistency between those two groups. Only biomarkers with consistent distribution were taken into multivariable analysis.

* *p*-value < 0.1 showed marginally statistically significance.

** *p*-value < 0.05 showed statistically significance.

Supplementary Table 4. Univariable and multivariable linear mixed-effect model for the association of combination molecular groups with tumor growth.

Molecular groups	No.	Univariable analysis			Multivariable analysis		
		Interaction coefficients	SE	*p*	Interaction coefficients	SE	*p*
IDH1 + *TERT* +1p/19q[a]							
Triple-positive	6	reference			reference		
TERTmt and *IDH1mt*	3	0.8	30.6	0.98	0.3	31.2	0.99
*IDH1*mt only	5	32.0	27.7	0.3	27.0	30.6	0.4
*TERT*mt only	12	73.0	23.2	0.002**	64.4	26.4	0.02**
Triple-negative	12	18.5	22.8	0.4	17.7	25.4	0.5
Other biomarkers							
WHOG (Grade IV vs III)	56 vs 55	27.5	9.8	0.005**	19.46	10.23	0.06
ATRX (high vs low)	14 vs 21	31.6	16.2	0.05*	23.22	17.02	0.17
Ki67 (high vs low)	55 vs 36	20.1	11.3	0.08*	16.64	11.18	0.90
MGMT (met vs non-met)	27 vs 10	-37.4	17.6	0.03**	-11.14	19.29	0.56
Other biomarkers							
IDH1 + *MGMT*[b]							
*IDH1*mt and *MGMT*met	14	reference			reference		
*IDH1*mt/*MGMT*met only	14	29.4	18.0	0.1	34.3	19.7	0.08*
IDH1 wt and non-*MGMT*met	9	61.0	20.9	0.004**	57.8	21.2	0.01**
Other biomarkers							
WHOG (Grade IV vs III)	56 vs 55	27.5	9.8	0.005**	16.57	10.48	0.12
ATRX (high vs low)	14 vs 21	31.6	16.2	0.05*	26.03	16.72	0.12
Ki67 (high vs low)	55 vs 36	20.1	11.3	0.08*	16.94	11.24	0.13
TERT (mt vs wt)	22 vs 19	52.4	25.7	0.04**	18.26	15.90	0.25
IDH1 + ATRX[c]							
IDH1mt + ATRX low	7	reference			reference		
IDH1mt + ATRX high	5	-10.4	25.9	0.7	-14.4	26.8	0.6
IDH1wt + ATRX low	14	3.3	21.5	0.9	-3.1	22.8	0.9
IDH1wt + ATRX high	9	61.9	23.7	0.01*	48.8	25.9	0.06*
Other biomarkers							
WHOG (Grade IV vs III)	56 vs 55	27.5	9.8	0.005**	19.05	10.13	0.06*
MGMT (met vs non-met)	27 vs 10	-37.4	17.6	0.03**	-27.14	17.64	0.13
Ki67 (high vs low)	55 vs 36	20.1	11.3	0.08*	17.96	11.27	0.11
TERT (mt vs wt)	22 vs 19	52.4	25.7	0.04**	11.14	15.14	0.46

Abbreviations: wt, wild type; mt, mutation type; met, methylation; SE, standard error.
* *p*-value < 0.1 showed marginally statistically significance.
** *p*-value < 0.05 showed statistically significance.

Supplementary Table 5. Comparison between linear mixed-effects models.

	DF	AIC	BIC	LogLik	MSE	R	LRStat	p
Previous Model a	4	1955.8	1970	-973.9	38.31	0.85	718.03	< 0.01*
Present Model	7	1243.8	1268.6	-614.88	1.16	0.99		
Formula								
Previous Model	$MTD_{ij} = \beta_0 + \beta_1 \times T_{ij} + \beta_2 \times iMTD + \alpha_{1i} + \alpha_{2i} \times T_{ij} + \varepsilon_{ij}$							
Present Model	$MTD_{ij} = \beta_0 + \beta_1 \times T_{ij} + \alpha_i + \varepsilon_{ij}$							

DF, degrees of freedom; AIC, Akaike information criterion; BIC, Bayesian information criterion; LogLik, maximized loglikelihood; LRStat, likelihood ratio test statistic.
a. Mandonnet E, Delattre JY, Tanguy ML, et al. Continuous growth of mean tumor diameter in a subset of grade II gliomas. Annals of neurology 2003;53:524-528.

Supplementary Table 6. Antibodies of immunohistochemical.

Antibody	Host species	Batch number	Clone	Dilution	Suppliers	Antigen retrieval
ATRX	rabbit	ZA-0016	pAbs	1:100	ZSGB-BIO	EDTA pH 8.0
GFAP	mouse	ZM-0118	UMAB129 mAb	1:100	ZSGB-BIO	EDTA pH 8.0
Olig-2	mouse	ZA-0561	EP112 mAb	1:100	ZSGB-BIO	EDTA pH 8.0
TOPO2	mouse	ZM-0245	OTI2D12 mAb	1:100	ZSGB-BIO	EDTA pH 8.0
P-170	mouse	ZM-0189	C494 mAb	1:100	ZSGB-BIO	EDTA pH 8.0
MMP-9	rabbit	ZA-0562	EP127 mAb	1:100	ZSGB-BIO	EDTA pH 8.0
GST-π	mouse	ZM-0110	OTI4B6 mAb	1:100	ZSGB-BIO	EDTA pH 8.0
Ki67	mouse	ZM-0167	MIB1 mAb	1:100	ZSGB-BIO	EDTA pH 8.0
MGMT	mouse	ZM-0461	UMAB56 mAb	1:100	ZSGB-BIO	EDTA pH 8.0
EGFR	rabbit	ZA-0505	EP22 mAb	1:100	ZSGB-BIO	EDTA pH 8.0
VEGF	rabbit	ZA-0509	PAbs	1:100	ZSGB-BIO	EDTA pH 8.0
PTEN	rabbit	ZA-0635	D4.3 mAb	1:100	ZSGB-BIO	EDTA pH 8.0
p53	mouse	ZM-0408	DO-7 mAb	1:100	ZSGB-BIO	EDTA pH 8.0

Abbreviations: pAbs, polyclonal antibody; mAb, monoclonal antibody; ZSGB-BIO, Zhongshan Gold Bridge Biotechnology; EDTA, Ethylene-diamine-tetraacetic acid.
