# Supplementary material for: Association of tumor growth rates with molecular biomarker status: a longitudinal study of high-grade glioma
Source: Aging (Albany NY). 2020 May 9;12(9):7908–26. doi: 10.18632/aging.103110 (PMC7244074; doi:10.18632/aging.103110)
Supplement: Supplementary Table 3 [file aging-12-103110-s001..docx]

**Supplementary Table 3** Univariable linear mixed-effect model for the association of molecular biomarkers with tumor growth.

| **Interaction term ^a^** |  | **Number** | |  | **Univariable analysis** | | | |
| --- | --- | --- | --- | --- | --- | --- | --- | --- |
| $I_{n}$ |  | Group 1 | Group 2 |  | $\beta_{n}$ | SE | *p^b^* | *p* of ANOVA |
| 1p loss |  |  |  |  |  |  |  |  |
| NOS vs non-NOS^c^ |  | 78 | 31 |  | 21.22 | 13.12 | 0.11 | — |
| Loss vs non-loss |  | 22 | 9 |  | -16.54 | 18.48 | 0.37 | 0.18 |
| 19q |  |  |  |  |  |  |  |  |
| NOS vs non-NOS |  | 78 | 31 |  | 21.22 | 13.12 | 0.11 | — |
| Loss vs non-loss |  | 18 | 13 |  | -16.48 | 17.04 | 0.33 | 0.17 |
| 1p19q |  |  |  |  |  |  |  |  |
| NOS vs non-NOS |  | 78 | 31 |  | 21.22 | 13.12 | 0.11 | — |
| LOH vs non-LOH |  | 22 | 9 |  | -17.27 | 18.31 | 0.35 | 0.17 |
| *IDH1* |  |  |  |  |  |  |  |  |
| NOS vs non-NOS |  | 62 | 47 |  | 9.29 | 9.31 | 0.32 | — |
| Mutation vs non mutation |  | 29 | 18 |  | -28.68 | 14.97 | **0.06*** | 0.10 |
| *MGMT* promoter |  |  |  |  |  |  |  |  |
| NOS vs non-NOS |  | 72 | 37 |  | 3.54 | 9.66 | 0.71 | — |
| Methylation vs non-methylation |  | 10 | 27 |  | -37.40 | 17.59 | **0.03**** | 0.10 |
| *TERT* promoter C228T |  |  |  |  |  |  |  |  |
| NOS vs non-NOS |  | 68 | 41 |  | 5.51 | 9.49 | 0.56 | — |
| Mutation vs non mutation |  | 23 | 18 |  | -1.94 | 15.02 | 0.90 | 0.84 |
| *TERT* promoter C250T |  |  |  |  |  |  |  |  |
| NOS vs non-NOS |  | 68 | 41 |  | 5.51 | 9.49 | 0.56 | — |
| Mutation vs non mutation |  | 37 | 4 |  | 52.36 | 25.69 | **0.04**** | 0.11 |
| *TERT* promoter (C250T/C228T) |  |  |  |  |  |  |  |  |
| NOS vs non-NOS |  | 41 | 68 |  | 5.51 | 9.49 | 0.56 | — |
| Mutation vs non mutation |  | 22 | 19 |  | 15.39 | 14.85 | 0.30 | 0.49 |
| ATRX |  |  |  |  |  |  |  |  |
| NOS vs non-NOS |  | 74 | 35 |  | 3.25 | 9.88 | 0.74 | — |
| Classification 1 ^d^ |  | 7 | 28 |  | 19.84 | 20.09 | 0.32 | 0.58 |
| Classification 2 |  | 13 | 22 |  | 14.75 | 16.74 | 0.38 | 0.64 |
| Classification 3 |  | 21 | 14 |  | 31.57 | 16.19 | **0.05*** | 0.14 |
| Classification 4 |  | 19 | 16 |  | 20.97 | 16.18 | 0.20 | 0.41 |
| Ki67 |  |  |  |  |  |  |  |  |
| NOS vs non-NOS |  | 18 | 91 |  | -4.71 | 12.31 | 0.70 | — |
| Classification 2 |  | 6 | 85 |  | 20.14 | 20.74 | 0.33 | 0.58 |
| Classification 3 |  | 36 | 55 |  | 20.14 | 11.32 | **0.08*** | 0.19 |
| Classification 4 |  | 85 | 6 |  | -14.70 | 21.20 | 0.49 | 0.73 |
| p53 |  |  |  |  |  |  |  |  |
| NOS vs non-NOS |  | 33 | 76 |  | 11.00 | 9.95 | 0.27 | — |
| Classification 1 |  | 15 | 61 |  | 6.63 | 14.32 | 0.64 | 0.49 |
| Classification 2 |  | 35 | 41 |  | 14.43 | 11.36 | 0.21 | 0.24 |
| Classification 3 |  | 50 | 26 |  | 12.76 | 11.86 | 0.28 | 0.31 |
| Classification 4 |  | 58 | 18 |  | 15.97 | 13.10 | 0.22 | 0.26 |
| VEGF |  |  |  |  |  |  |  |  |
| NOS vs non-NOS |  | 55 | 54 |  | 18.26 | 9.13 | **0.05*** | — |
| Classification 1 |  | 21 | 33 |  | -11.49 | 14.00 | 0.41 | 0.10 |
| Classification 2 |  | 30 | 24 |  | -5.06 | 13.73 | 0.71 | 0.13 |
| Classification 3 |  | 44 | 10 |  | -2.71 | 17.50 | 0.88 | 0.14 |
| Classification 4 |  | 49 | 5 |  | -0.55 | 23.14 | 0.98 | 0.14 |
| EGFR |  |  |  |  |  |  |  |  |
| NOS vs non-NOS |  | 39 | 70 |  | 13.08 | 9.58 | 0.17 | — |
| Classification 1 |  | 9 | 61 |  | -3.92 | 17.28 | 0.82 | 0.39 |
| Classification 2 |  | 14 | 56 |  | 10.58 | 14.59 | 0.47 | 0.31 |
| Classification 3 |  | 27 | 43 |  | 17.55 | 11.94 | 0.14 | 0.13 |
| Classification 4 |  | 38 | 32 |  | 17.99 | 11.81 | 0.13 | 0.12 |
| MGMT |  |  |  |  |  |  |  |  |
| NOS vs non-NOS |  | 62 | 47 |  | 17.43 | 9.33 | **0.06*** | — |
| Classification 1 |  | 16 | 31 |  | -4.77 | 15.10 | 0.75 | 0.17 |
| Classification 2 |  | 26 | 21 |  | -16.83 | 14.56 | 0.25 | **0.09*** |
| Classification 3 |  | 40 | 7 |  | -5.30 | 19.74 | 0.79 | 0.17 |
| Classification 4 |  | 45 | 2 |  | -24.63 | 33.07 | 0.46 | 0.14 |
| MMP9 |  |  |  |  |  |  |  |  |
| NOS vs non-NOS |  | 41 | 68 |  | 18.78 | 9.42 | **0.05*** | — |
| Classification 1 |  | 32 | 36 |  | -6.19 | 11.91 | 0.60 | 0.12 |
| Classification 2 |  | 51 | 17 |  | 17.56 | 13.53 | 0.20 | **0.06*** |
| Classification 3 |  | 60 | 8 |  | 22.63 | 18.44 | 0.22 | 0.06 |
| Classification 4 |  | 64 | 4 |  | 12.15 | 24.23 | 0.62 | 0.12 |
| PTEN |  |  |  |  |  |  |  |  |
| NOS vs non-NOS |  | 55 | 54 |  | 18.26 | 9.13 | **0.05*** | — |
| Classification 1 |  | 13 | 41 |  | -7.01 | 15.90 | 0.66 | 0.13 |
| Classification 2 |  | 27 | 27 |  | -7.02 | 13.38 | 0.60 | 0.12 |
| Classification 3 |  | 40 | 14 |  | -17.48 | 15.05 | 0.25 | **0.07*** |
| Classification 4 |  | 46 | 8 |  | -16.63 | 18.56 | 0.37 | **0.09*** |
| GFAP |  |  |  |  |  |  |  |  |
| NOS vs non-NOS |  | 55 | 54 |  | -0.37 | 9.30 | 0.97 | — |
| Classification 2 |  | 40 | 14 |  | -15.90 | 15.08 | 0.29 | 0.57 |
| Classification 3 |  | 43 | 11 |  | -12.03 | 16.40 | 0.46 | 0.76 |
| Classification 4 |  | 47 | 7 |  | -15.28 | 19.24 | 0.43 | 0.73 |
| Olig2 |  |  |  |  |  |  |  |  |
| NOS vs non-NOS |  | 63 | 46 |  | 7.01 | 9.41 | 0.46 | — |
| Classification 1 |  | 5 | 41 |  | -26.24 | 25.06 | 0.30 | 0.43 |
| Classification 2 |  | 14 | 32 |  | 11.61 | 15.88 | 0.47 | 0.58 |
| Classification 3 |  | 44 | 2 |  | -21.16 | 34.64 | 0.54 | 0.63 |
| Classification 4 |  | 45 | 1 |  | -25.38 | 46.48 | 0.59 | 0.65 |
| TOPO2 |  |  |  |  |  |  |  |  |
| NOS vs non-NOS |  | 54 | 55 |  | 11.63 | 9.23 | 0.21 | — |
| Classification 1 |  | 4 | 51 |  | 2.94 | 26.43 | 0.91 | 0.45 |
| Classification 2 |  | 12 | 43 |  | 23.81 | 16.56 | 0.15 | 0.16 |
| Classification 3 |  | 48 | 7 |  | -23.37 | 19.69 | 0.24 | 0.22 |
| Classification 4 |  | 54 | 1 |  | -12.45 | 48.85 | 0.80 | 0.44 |
| GST-π |  |  |  |  |  |  |  |  |
| NOS vs non-NOS |  | 65 | 44 |  | 16.69 | 9.43 | **0.08*** | — |
| Classification 1 |  | 7 | 37 |  | -15.15 | 20.90 | 0.47 | 0.16 |
| Classification 2 |  | 20 | 24 |  | -12.06 | 15.07 | 0.42 | 0.16 |
| Classification 3 |  | 32 | 12 |  | -13.56 | 16.53 | 0.41 | 0.15 |
| Classification 4 |  | 40 | 4 |  | 18.76 | 25.66 | 0.47 | 0.16 |
| P170 |  |  |  |  |  |  |  |  |
| NOS vs non-NOS |  | 55 | 54 |  | 18.26 | 9.13 | **0.05*** | — |
| Classification 1 |  | 21 | 33 |  | -9.41 | 13.80 | 0.50 | 0.11 |
| Classification 2 |  | 25 | 29 |  | -10.56 | 13.46 | 0.43 | 0.10 |
| Classification 3 |  | 38 | 16 |  | -8.81 | 14.43 | 0.54 | 0.12 |
| Classification 4 |  | 43 | 11 |  | -1.85 | 16.55 | 0.91 | 0.14 |
| *IDH1* + *TERT* +1p/19q |  |  |  |  |  |  |  |  |
| NOS vs non-NOS |  | 38 | 71 |  | 4.50 | 9.63 | 0.64 | — |
| Triple-positive |  | 6 |  |  | reference | |  | **0.01**** |
| *TERTmt* and *IDH1mt* |  |  | 3 |  | 0.77 | 30.63 | 0.98 |  |
| *IDH1*mt only |  |  | 5 |  | 32.04 | 27.65 | 0.25 |  |
| *TERT*mt only |  |  | 12 |  | 73.04 | 23.24 | **0.002**** |  |
| Triple-negative |  |  | 12 |  | 18.54 | 22.82 | 0.42 |  |
| *IDH1 + MGMT* |  |  |  |  |  |  |  |  |
| NOS vs non-NOS |  | 37 | 72 |  | 3.54 | 9.66 | 0.71 | — |
| *IDH1*mt and *MGMT*met |  | 14 |  |  | reference | |  | **0.03**** |
| *IDH1*mt/*MGMT*met only |  |  | 14 |  | 29.36 | 17.95 | 0.10 |  |
| *IDH1* wt and non-*MGMT*met |  |  | 9 |  | 60.96 | 20.92 | **0.004**** |  |
| *IDH1 +* ATRX |  |  |  |  |  |  |  |  |
| NOS vs non-NOS |  | 35 | 74 |  | 3.25 | 9.88 | 0.74 | — |
| *IDH1mt +* ATRX low |  | 7 |  |  | reference | |  | **0.02**** |
| *IDH1mt+*ATRX high |  |  | 5 |  | -10.35 | 25.87 | 0.69 |  |
| *IDH1wt +* ATRX low |  |  | 14 |  | 3.30 | 21.53 | 0.88 |  |
| *IDH1wt +* ATRX high |  |  | 9 |  | 61.94 | 23.74 | **0.01**** |  |
| *IDH1*mt + 1p/19q LOH +ATRX |  |  |  |  |  |  |  |  |
| NOS vs non-NOS |  | 5 | 104 |  | 26.62 | 16.72 | 0.11 | — |
| ATRX low vs high |  | 2 | 3 |  | 7.98 | 30.56 | 0.79 |  |

Abbreviation: SE, standard error; ANOVA, analysis of variance; LOH, 1p/19q loss of heterozygosity; NOS, not otherwise specified; wt, wild type; mt, mutation type; met, methylation; SE, standard error.

a. Evaluation of acceleration effects of molecular biomarkers combined with significant clinical biomarkers in LME:

$${MTD}_{ij}=\beta_{0}+\beta_{1}{\times T}_{ij}+\beta_{2}\times iMTD+\beta_{3}\times(WHO grades\times T_{ij})+\beta_{3}\times WHO grades+\beta_{n}{\times(I}_{n}\times T_{ij})+\beta_{n+1}{\times I}_{n}+\alpha_{1i}+\alpha_{2i}\times T_{ij}+\varepsilon_{ij}$$

b. The *p* value indicates the acceleration effects of group 2 compared to group 1, *p* value of ANOVA indicates the whole acceleration effects of biomarkers including NOS type on tumor growth.

c. For biomarkers with unknown or NOS group, we first evaluate the NOS group with the non-NOS group to figure out if the subgroups were distributed inconsistency between those two groups. Only biomarkers with consistent distribution were taken into multivariable analysis.

d. Biomarkers were classified according to different cutoffs of staining scores: low expression vs high expression, classification 1, score 0 vs 1-4; classification 2, score 0/1 vs 2-4; classification 3, score 0-2 vs 3/4; classification 4, score 0-3 vs 4.

* *p*-value < 0.1 showed marginally statistically significance.

** *p*-value < 0.05 showed statistically significance.
